# When Prostate Cancer Circulates in the Bloodstream

**DOI:** 10.3390/diagnostics5040428

**Published:** 2015-10-29

**Authors:** Virginie Vlaeminck-Guillem

**Affiliations:** 1 Cancer Research Centre of Lyon, U1052 INSERM, CNRS 5286, Léon Bérard Centre, Lyon I University, 28 rue Laennec, Lyon 69008, France; 2 Medical Unit of Molecular Oncology and Transfer, Department of Biochemistry and Molecular Biology, University Hospital of Lyon-Sud, Hospices Civils of Lyon, Lyon 69008, France

**Keywords:** prostate cancer, circulating biomarker, diagnosis, tumor aggressiveness, metastasis, circulating tumor cells, extracellular vesicles, exosomes, free cell DNA, miRNA

## Abstract

Management of patients with prostate cancer is currently based on imperfect clinical, biological, radiological and pathological evaluation. Prostate cancer aggressiveness, including metastatic potential, remains difficult to accurately estimate. In an attempt to better adapt therapeutics to an individual (personalized medicine), reliable evaluation of the intrinsic molecular biology of the tumor is warranted, and particularly for all tumor sites (primary tumors and secondary sites) at any time of the disease progression. As a consequence of their natural tendency to grow (passive invasion) or as a consequence of an active blood vessel invasion by metastase-initiating cells, tumors shed various materials into the bloodstream. Major efforts have been recently made to develop powerful and accurate methods able to detect, quantify and/or analyze all these circulating tumor materials: circulating tumors cells, disseminating tumor cells, extracellular vesicles (including exosomes), nucleic acids, *etc.* The aim of this review is to summarize current knowledge about these circulating tumor materials and their applications in translational research.

## 1. Introduction

Prostate cancer (PCa) is the most frequent cancer in men of Western countries. PCa can be diagnosed at, or will evolve towards, a metastatic state, bone metastases being by far the most frequent sites for metastatic grafts. PCa cells are indeed initially hormone-sensitive or even hormone-dependent for their growth. A standard treatment is therefore to induce androgen deprivation (castration) through surgical or pharmacological means. Hormone-sensitive PCa temporarily regresses under androgen deprivation but castration-resistant cancer cells eventually survive and grow unequivocally until patient’s death.

The process by which a tumor spreads to a distant site to form a metastasis is multistage and complex. Several steps are needed from the escape from the primary tumor and intravasation, towards extravasation and successful implantation in the host tissue. The obligatory intermediate step is circulation of tumor cells in the blood. For the clinician, the direct application of this concept is the presumptive interest in detecting circulating tumor cells, at least to identify a potential ongoing metastatic process. However, this concept can even be extended to all other circulating materials that escape from the primary tumor and circulate in the blood. The PSA (prostate specific antigen), as the classical and universally used PCa biomarker, is a demonstrative example. Abnormally produced by the tumor because of a strong alteration in the prostate tissue organization, it can be detected and measured in the serum as a means to detect PCa, to evaluate PCa aggressiveness and to monitor PCa under or after treatment. Many other putative circulating proteins (including free and precursor isoforms of PSA) have been evaluated or are currently under evaluation to complement the poor PSA specificity in managing PCa patients. This topic will not be approached in this review. The aim of the present review is to describe all other circulating tumor materials that can escape from the primary and/or secondary tumors (Table 1), such as the circulating tumor cells (CTCs) and their bone marrow counterparts (the so-called disseminated tumor cells, DTC), as well as the circulating nucleic acids (DNAs, mRNAs, microRNAs), whether they circulate free from cells or as components of circulating microvesicles (particularly exosomes). By comparison with the PSA, which clearly constitutes an imperfect witness of tumor biology, these cellular and non-cellular circulating materials have a clear biological signification as markers of PCa aggressiveness and/or actors of the metastatic process.

## 2. Circulating and Disseminating Tumor Cells

### 2.1. CTC Biology

Circulating tumor cells are defined as intact cells that progress into blood vessels from primary or secondary tumor deposits. They are thought to finally colonize at distant sites and form metastases. A rat model study allowed estimation that solid tumors daily shed 3.2–4.1 × 10^6^ CTCs per gram of tissue [1]. The CTC abundance is, however, estimated as one CTC in 10^6^–10^7^ leukocytes in one milliliter of peripheral blood of cancer patients [2,3]. This discrepancy is explained by the supposed short half-life, as suggested by a precipitous postoperative decline (<24 h) after surgery for localized PCa [4] and evaluated between 1 and 2.4 h for mammary cancer cells [5]. This suggests a constant renewing of CTCs in the blood.

**Table 1:** The various circulating tumor materials, their properties, their methods of detection and their applications in the field of prostate cancer.

Circulating Tumor Materials	How do They Circulate in the Bloodstream	Main Properties	How to Detect Them	Main Results in Prostate Cancer
Circulating tumor cells (CTCs) 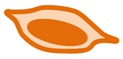	Isolated cells Cell clusters Cell fragments	Epithelial-to-mesenchymal transition Plasticity (organ mimicry) Resistance to anoikis Immune cell escape Ability to initiate metastases	3 steps: Enrichment Tumor cell staining or oncogene probing Detection The FDA-approved CellSearch system is the most used method. Can be evaluated: CTC count (cutoff 5 CTCs/7.5 mL) Specific expression patterns Functional properties (ex: ELISPOT)	Poor ability of CTC count to diagnose early PCa Inconstant correlations between CTC counts and tumor burden, pN status, pM status Frequent correlation between CTC count and overall survival Evaluation of CTC count under treatment would be predictive of disease progression CTC count could be used as a surrogate marker of survival in clinical trials
Extracellular vesicles (EVs) 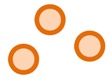	Apoptotic bodies Microvesicles Exosomes	Intercellular trafficking Biologically significant cargo: proteins, lipids nucleic acids Ability to influence the biology of target cells	Centrifugation-based purification With difficulties	Mostly exosomes have been studies PCa cells produce exosomes Numerous specific or high throughput analyses of exosome contents have been performed No immediate transfer into clinical practice
DNAs 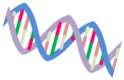	Cell-free Mostly short fragments (apoptotic release) In plasma or serum	Reliable markers of intrinsic tumor biology	Mostly PCR-based methods Whole sequencing No standardization to date Can be evaluated: Whole DNA levels Targeted genetic alterations Whole genetic alterations DNA integrity Epigenetic events (methylation)	Extreme variations in the design of the published studies Contradictory results when evaluated for either diagnostic or prognostic purposes High potential interest to predict response to treatment and to personalize the treatment
microRNAs (miRs) 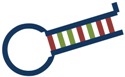	Mostly as exosomal constituents Bound to high-density lipoproteins Bound to Ago2 protein	Strong association between miR and exosomal maturation processes Ability to influence the biology of target cells	Mostly RT-PCR-based methods Whole sequencing	Deregulation of several miRs has been associated with PCa risk, aggressiveness, staging and outcome miR-141 is one the most studied miRs No immediate transfer into clinical practice

CTCs can deposit and remain as individuals within the bone marrow. They are then called disseminated tumor cells (DTCs). In the bone marrow, DTCs can alternatively stay in a dormancy state [6,7], sometimes for many years [8], form micro- and then macrometastases, or return into the blood to fuel the CTC compartment [5]. CTCs can even re-infiltrate the primary tumor or the primary site cured by an effective treatment to induce tumor progression or recurrence, respectively [9,10,11]. Survival of CTCs in the blood circulation is likely to be obtained because of specific cell properties. This depends on whether this intravasation is active or passive [8]. Passive entry is the result of a vessel leakage by a growing tumor and external forces (friction, surgical manipulation, *etc*.) that detach cells. In this case, shed cells are *mobile* without requiring specific features. Active entry requires *motile* cells, that are cells with specific abilities to detach from other tumor cells, survive free of them, progress in the host tissue toward a blood vessel and then into the vessel lumen. This underlies that CTC population is heterogeneous, ranging from metastatic founder cells (called metastases initiating cells, MICs) with specific cell properties [12] to poorly aggressive cells without any specific ability to survive into the blood.

One of the main supposed traits of motile CTCs is the ability to engage in an epithelial-to-mesenchymal transition (EMT) process. The EMT concept for CTCs has been demonstrated for several cancers, including PCa, as gains in expression of mesenchymal markers such as vimentin, N-cadherin or O-cadherin [13,14]. Chen *et al.* [15] examined EMT-specific profiles in CTCs from eight patients with advanced PCa using multiplex RT-PCR. Heterogeneous expression patterns were observed with globally a decrease in epithelial marker expression. Interestingly, an increase in EMT-related genes was more frequently observed in CTCs of castration-resistant PCas as compared to hormone-sensitive PCas [15], consistently with the aggressiveness-favoring hypothesis of EMT. The engagement towards the EMT process should, however, not be definitive: when cells are arrested in a mesenchymal state, they can easily escape from the primary tumor and progress within the tissue towards the blood vessels but can hardly implant in a metastatic site [16,17,18,19]. Intermediate states, associating both epithelial and mesenchymal markers and therefore reflecting cell plasticity, provide better advantage in metastasis formation. Such a cell plasticity is a landmark of cancer stem cells, a term which is probably another way to define CTCs with MIC-like properties [20,21,22,23]. Indeed, CTCs from PCa have been demonstrated to express CD133 [14] or ALDH1 [24], both markers of cell stemness. Moreover, a kind of “organ mimetic phenotype” [25] should be acquired by the CTCs, which allows specific homing to a targeted tissue. In this setting, identifying this organ-specific phenotype would be of great clinical importance when isolating CTCs from blood in a cancer patient: guided and efficient imaging could be performed. For PCa, which usually evolves to bone metastases, cell plasticity allows metastatic cells to express bone markers in order to better implant in bone tissue. To our knowledge, no study specifically explored markers of osteomimicry in CTCs originating from PCa although it would deserve to evaluate whether CTCs already express these markers.

Other properties are required for CTC survival in the blood circulation. CTCs have to resist to anoikis (anchorage-dependent cell death). Overexpression of anti-apoptotic proteins or activation of specific pathways have been described in CTCs, such as Bcl-2 overexpression [26] or activation of the tropomyosin-related kinase B (TrkB) [27]. In the blood, CTCs are also challenged by the host’s immunological defenses and should develop mechanisms to escape immune cells. One of these mechanisms is the upregulation of CD47 which prevents CTCs from macrophage and dendritic cell attacks [28].

Overall, only few of the millions of tumor cells continuously shed through the body are able to reach a distant site, survive in a dormant state, evade the immune system or even systemic cytolytic therapy given for the cancer and eventually form a macrometastasis. Some former studies suggested that 0.01% of CTCs ultimately can produce a single bone metastasis, and at least 104 circulating tumor cells are required for the development of a metastatic focus [29,30]. More recently, Carvalho *et al.* [31] sampled CTCs from men with castration-resistant PCas and implanted them in immuno-compromised mice without obtaining overt metastases. The number of available CTCs is likely to be a determinant factor: injection of CTCs obtained from metastatic breast into bone marrow of immuno-compromised mice induced macrometastases only if CTCs were more numerous than 1000 per 7.5 mL [21]. However, the availability of a specific genomic program is also of primary importance for CTCs engaged in the metastatic process: in the same study, a specific expression pattern associated with the ability to form metastases was defined (CD45^−^/EpCAM^+^/CD44^+^/CD47^+^/cMet^+^), probably corresponding to MIC’s expression pattern [21].

### 2.2. Methods to Detect CTCs and DTCs and Their Application to Prostate Cancer

Until recently, CTCs and DTCs could be only detected through the indirect detection of RNA and protein markers in a blood sample or in a bone marrow aspirate supposed to be enriched in CTCs or DTCs. Wood *et al.* [32] found DTCs in bone marrow of patients with localized PCa as soon as 1994. Melchior *et al.* [33] confirmed PSA positive RT-PCR in peripheral blood and bone marrow of patients with localized and metastatic PCa in 1997, followed by several studies in the late 90s [34,35,36]. These methods were associated with major drawbacks including the strong inter-laboratory variability, the lack of evidence that the RNAs and proteins were really obtained from CTCs or DTCs (illegitimate expression by blood cells), and the lack of evidence that the originating cells were viable or even intact. Direct detection of CTCs was therefore expected and obtained for PCa in the early 2000s [37,38]. The former study used CD45-based immunomagnetic separation (negative enrichment by removing hematopoietic cells) followed by cytokeratin and PSMA-based immunohistochemistry in 25 patients with metastatic PCa and 63 healthy controls [37]. No prostate circulating cell was observed in healthy controls while they were found in 18 of the PCa patients (72%) [37]. The latter study used epithelial cell adhesion molecule (EpCAM)-based immunomagnetic separation (positive enrichment) followed by fluorescence-activated cell sorting (FACS) in 20 PCa patients and 22 healthy control men. Although circulating epithelial cells were found in healthy controls, significantly-higher CTC counts were obtained in the 10 patients with localized PCa and in the 10 patients with metastatic PCa [38]. Interestingly, there was a trend for a correlation between CTC count and disease progression, without correlation with serum PSA evolution, suggesting that CTC enumeration could provide valuable additional information [38].

Numerous CTC isolation and capture techniques have been reported so far, but only one method, CellSearch (Veridex), is cleared by the FDA for use for metastatic prostate, breast and colon cancers. It has indeed been analytically validated with good reproducibility, low intra-patient and inter-laboratory variability [39,40]. The assay enriches cells on the basis of antibodies to EpCAM conjugated to magnetic beads, and are further classified as CTCs on the basis of morphologic limits (round or oval morphology; size >5 μm), of rigorous criteria for staining for cytokeratin (CK-6, 8, 18), of displaying a nucleus [positive staining for 4′,6-diamidino-2-phenylindole (DAPI)], and of excluding white blood cell (negative staining for CD45). Many other technologies have been proposed and/or are currently in development to increase CTC detection rates and/or isolate viable cells that can be evaluated for their properties and, particularly, their aggressive behavior [13,41,42]. All these techniques include a first enrichment step, a second tumor cell staining or oncogene probing step and a third detection step [43]. Enrichment is an unavoidable crucial step because of the relatively low number of CTCs among other circulating cells. However, it is also a selecting step that isolates and identifies CTCs based on a certain set of characteristics: since CTCs are highly heterogeneous, several CTCs are inevitably ignored. The CellSearch system for example selects CTCs based on the expression of an epithelial marker (EpCAM) although molecular phenotyping of CTCs allowed demonstration that CTCs frequently lost epithelial characteristics because of EMT [3]. The use of other capture antibodies is therefore warranted and could be directed against other antigens specific for CTCs such as mesenchymal or stemness ones [44]. Mesenchymal-like CTC subpopulations are, however, likely to be difficult to identify in the hematopoietic environment, which is also of mesenchymal origin [45]. Another pitfall is the potential detection of prostate derived, circulating cancer-associated fibroblasts (CAFs) as suggested by a recent study that used the CellSearch technology and capture antibodies directed against mesenchymal markers [46]. Techniques that detect CTCs are therefore always a compromise between sensitivity and purity: detect all CTC subpopulations without detecting other circulating cells. Detection of all CTCs may be also hampered by the low volume of blood evaluated (7.5 mL for the CellSearch system).

### 2.3. Diagnostic and Prognostic Value of CTC Enumeration in Prostate Cancer

Techniques for direct detection of CTCs have been early applied to PCa patients. As a pioneer example, Meye *et al.* [47] found epithelial (cytokeratin-positive) cells in epithelial cell-enriched, leukocyte-depleted peripheral blood samples of 23/60 (38%) PCa patients. Interestingly cytokeratin-positive cells were not detected in PCa-free samples [47]. Whether CTC enumeration could be used for PCa diagnosis (whatever the clinical stage) was mostly questioned following a hopeful study that detected CTCs in several cancers including localized PCas [48]. Those patients even had higher CTC numbers than patients with metastatic PCa. Other studies however provided various results (Table 2), depending on the technique used and whether the judgment criterion was test positivity (presence of CTCs or not) [41] or CTC counts. In the latter case, similar CTC counts were usually observed in patients with localized PCa and healthy controls (Table 2) [4,49,50,51], suggesting the poor ability of CTC enumeration to diagnose early PCas.

**Table 2:** Studies evaluating the ability of CTC enumeration to diagnose prostate cancer.

References	Technique for CTC Detection	Prostate Cancer Group	Control Group	Results
[37]	Immunomagnetic separation followed by cytokeratin and PSMA IHC	*n* = 25 patients with mPCa	*n* = 63 healthy controls	No CTC in healthy controls CTCs in 72% of the PCa patients
[38]	Immunomagnetic separation followed by FACS	*n* = 10 patients with lPCa *n* = 10 patients with mPCa	*n* = 22 healthy controls	Higher CTC counts in patients with lPCa or mPCa than in healthy controls
[47]	Immunomagnetic separation followed by cytokeratin IHC	*n* = 60 PCa, most of them before RP	*n* = 20 healthy controls	No CTC in healthy controls CTCs in 38% of the PCa patients
[52]	Immunomagnetic separation followed by RT-PCR (PSA)	*n* = 284 PCa including: 138 patients before RP 31 patients with post-RP recurrence 37 patients under ADT 11 patients dead from PCa 67 patients with no evidence of disease after treatment	*n* = 52 healthy controls *n* = 51 men with elevated PSA levels and negative prostate biopsies or TURP *n* = 32 patients with other cancers	CTCs in: none of the healthy controls and other cancer patients 4% of the patients with elevated PSA levels and negative prostate biopsies or TURP 24% of the patients before RP 51% of the patients with progressive PCa 9% of the patients with no evidence of disease <5 years after treatment None of the patients with no evidence of disease >5 years after treatment
[41]	Elispot	*n* = 24 patients with lPCa (12 before and 12 after treatment) *n* = 24 patients with mPCa	*n* = 31 patients with BPH or acute prostatitis *n* = 35 patients without prostate pathology *n* = 8 healthy controls	No CTC in non-PCa patients Test more frequently positive in the 24 mPCa (83%) than in the 12 lPCa before treatment (42%) No CTC in the 12 lPCa after treatment
[53]	CellSearch	*n* = 84 patients with advanced PCa (130 samples)	*n* = 39 healthy controls	<2 CTCs/7.5 mL in healthy controls ≥2 CTCs/7.5 mL in 62% of the 130 cancer samples
[54]	Immunomagnetic separation followed by RT-PCR (PSA)	*n* = 371 PCa including: 183 patients before RP 34 patients with post-RP recurrence 64 patients under ADT 90 patients with no evidence of disease after treatment	*n* = 78 healthy controls *n* = 63 men with elevated PSA levels and negative prostate biopsies or TURP	CTCs in: none of the healthy controls 3% of the patients with elevated PSA levels and negative prostate biopsies or TURP 20% of the patients before RP 46% of the patients with progressive PCa 10% of the patients with no evidence of disease <1 year after treatment None of the patients with no evidence of disease >1 year after treatment
[51]	CellSearch	*n* = 97 patients prior to RP	*n* = 25 men with elevated PSA levels and negative prostate biopsies	CTCs detected in 21% *vs.* 20% (PCa *vs.* control groups)
[4]	CTC-Chip	*n* = 55 patients (including 19 patients with lPCa)	*n* = 17 healthy controls	CTCs detected in 8/17 healthy controls (max: 10 CTCs/7.5 mL) 8/19 patients with lPCa had ≥14 CTCs
[55]	CTC-Chip (herringbone Chip)	*n* = 15 patients with mPCa	*n* = 10 healthy controls	0 to 8 CTCs/7.5mL on healthy controls ≥12 CTCs in 14 of the mPCa patients
[50]	CellSearch	*n* = 26 patients with lPCa	*n* = 30 healthy controls	3 healthy controls with 1 CTC/7.5 mL No difference for the mean CTC counts
[56]	CellSearch	*n* = 26 patients with PCa and biochemical recurrence after RP	*n* = 7 healthy controls	No CTC in healthy controls CTCs in 73% of the PCa patients
[49]	CellSearch	*n* = 20 patients with lPCa and high recurrence risk	*n* = 15 healthy controls	No difference for the mean CTC counts

ADT: androgen-deprivation therapy; BPH: benign prostate hyperplasia; CTC: circulating tumor cell; IHC: immunohistochemistry; FACS: fluorescence-activated cell sorting; lPCa: localized prostate cancer; mPCa: metastatic prostate cancer; PCa: prostate cancer; PSA: prostate-specific antigen; RP: radical prostatectomy; TURP: trans-urethral resection of the prostate.

Whether CTC enumeration could be useful in distinguishing patients with localized PCa and those with metastatic PCa is of major importance to better adapt treatment, mostly because imaging lacks accuracy, nomograms are still under evaluation and the only surgical means (diagnostic lymphadenectomy) may not necessarily reflect the hematogenous nature of the disease [57]. Several studies indeed suggested that higher CTC counts are correlated with the tumor burden [52], the presence of lymph node metastases [50], the presence of distant metastases (in particular, bone metastases comparing to soft tissue metastases) [41,49,50,58,59,60]. Other correlations have been variably reported with clinical or biological markers known to predict disease progression in metastatic PCas (PSA, alkaline phosphatase, lactate dehydrogenase, hemoglobin, *etc*.) (Table 3).

These correlations were inconsistently demonstrated and transfer to clinical practice remains elusive. It is, however, worth noting that correlation with overall survival was observed in all the published studies, particularly in patients with metastatic PCas, and often in a way independent from known survival predictors (Table 4). Furthermore, when tested, the CTC count was a better predictor of overall outcome than the usual marker PSA [61]. For this question, the usual cutoff is 5 CTCs/7.5 mL.

Sequential count of CTCs in the same patient allows determination of CTC evolution, particularly under treatment. Several studies reported a decrease in the CTC detection rate or count [4,24,41,56,59,62,63]. Evolution of CTC levels was suggestive of a disease progression under treatment [24,58], even anticipating the results of the radiological explorations [64]. As expected, this evolution also proved to be a predictor of survival [62,65] with a growing risk of death under the following scenarios: persistence of a favorable CTC count, transition from an unfavorable to a favorable count, transition from a favorable to an unfavorable count and persistence of an unfavorable count [61,66,67,68,69,70]. In this setting, the CTC count decline under treatment was a better predictor of overall survival than the PSA decline [66,69]. Overall, CTC enumeration is a strong indicator of disease progression, both spontaneously and under treatment. As such, it fulfills most of the criteria to be used as a surrogate of overall survival in clinical trials [61,63,65,68]. CTC counts were then used as an intermediary endpoint to assess treatment efficacy (along with PSA decline or ECOG status) in a phase II trial that included 58 men receiving abiraterone acetate after docetaxel failure for metastatic CRPC and did not evaluate overall survival as an endpoint criteria [71].

**Table 3:** Correlations between CTC enumeration and clinical, biological, radiological or pathological prognosis factors.

Reference	Patients	Methods	Correlations						
			Positive Correlation	Negative Correlation	No Correlation	Treatment		Metastases Status	
						Lower CTC Count	Higher CTC Count	Lower CTC Count	Higher CTC Count
[38]	*n* = 10 mPCa	IM/FACS	Disease progression	-	-	-	-	-	
[47]	*n* = 60 PCa	IM/IHC	Tumor stage	-	-	-	-	-	
[52]	*n* = 284 PCa	IM/RT-PCR	Tumor burden	-	-	-	-	-	
[53]	*n* = 85 advanced PCa	CellSearch	PSA; AP; LDH	Hb Creatinine	Gleason score	Androgen-depletion alone	Androgen-depletion and chemotherapy	No difference	
[41]	*n* = 24 lPCa and 24 mPCa	Elispot	-	-	-	For the lPCa:		lPCa	mPCa
						No treatment	Treatment		
[72]	*n* = 41 CRPC	IM/FACS	PSA; AP	Age	LDH; Hb; ECOG status	No difference when comparing the number of previous treatments		-	-
[58]	*n* = 112 mPCa	CellSearch	PSA; Bone metastases burden	-	-	No chemotherapy	Chemotherapy	Soft tissue only	Bone or Bone and soft tissue
[54]	*n* = 371 PCa	IM/RT-PCR	PSA	-	Gleason score; Tumor stage; Resection margins; pN status	-	-	-	-
[4]	*n* = 19 lPCa	CTC-Chip	-	-	PSA; Gleason score; Tumor size; Extracapsular extension; pN status; Perineural invasion; Resection margins	-	-	-	-
[24]	*n* = 16 CRPC	Adnagen	Radiological response	-	-	No disease progression under treatment	Disease progression under treatment	-	-
[56]	*n* = 26 PCa with rising PSA after RP	CellSearch	-	-	PSA	-	-	-	-
[50]	*n* = 26 lPCa	CellSearch	pN status; PSA; Tumor size	-	Gleason score	-	-	No metastases	Metastasis
[64]	*n* = 21	Adnagen	Disease progression	-	-	Disease controlled under treatment	Disease not controlled under treatment	-	-
[60]	*n* = 202 PCa	CellSearch	PSA; Gleason score	-	-	Non androgen-depleted	Androgen-depleted	Lymph node only	Bone or Bone and lymph node
[49]	*n* = 41 CRPC	CellSearch	AP; LDH	Hb;PSADT	PSA; Calcemia; Bone metastatic burden	-	-	Soft tissue only	Bone or Bone and soft tissue

AP: Alkaline phosphatase; CTC: circulating tumor cell; FACS: fluorescence-activated cell sorting; Hb: hemoglobin; IHC: immunohistochemistry; IM: immunomagnetic separation; LDH: lactate dehydrogenase; lPCa: localized PCa; mPCa: metastatic PCa; PCa: prostate cancer; PSA: prostate specific antigen; RP: radical prostatectomy.

**Table 4:** Results of the studies that evaluated CTC count as a predictor of overall survival.

Reference	Clinical Situation	Number of Patients	Methods	Use of CTC Count	CTC count as Predictor of overall Survival	Remark
[73]	mPCa	*n* = 37	IM/FACS	Continuous variable	Yes Independently	Similar results for a subgroups of 26 CRPC
[72]	CRPC	*n* = 41	IM/FACS	Binary variable (cutoff: 1.8)	Yes	Cutoff 1.8 was considered as the best cutoff to separate patients with favorable or unfavorable survival outcome
[58]	mPCa	*n* = 112	CellSearch	Continuous variable	Yes Independently	-
[69]	CRPC	*n* = 231	CellSearch	Binary variable (cutoff: 5)	Yes Independently	Part of the IMMC38 trial CTC counts at 2–5 weeks were also predictors
[74]	CRPC before docetaxel	*n* = 164	CellSearch	Continuous variable	Yes Independently	Part of the IMMC38 trial (same patients than [69]) CTC counts at 4, 8 and 12 weeks were also predictors
[62]	CRPC	*n* = 51	CellSearch	-	Yes	-
[70]	CRPC	*n* = 64	CellSearch	Binary variable (cutoff: 5)	Yes Independently	-
[68]	CRPC	*n* = 119	CellSearch	Binary variable (cutoff: 5)	Yes Independently	-
[75]	CRPC	*n* = 100	CellSearch	Binary variable (cutoff: 4)	Yes Independently	Cutoff 4 was considered as the best cutoff to separate patients with favorable or unfavorable survival outcome
[76]	CRPC before docetaxel	*n* = 179	CellSearch	Continuous variable	Yes	Part of the IMMC38 trial (same patients than [69])
[67]	CRPC	*n* = 76	CellSearch	Binary variable (cutoff: 5)	Yes Independently	-
[59]	CRPC	*n* = 162	CellSearch	Binary variable (cutoff: 5)	Yes	Part of the IMMC38 trial (same patients than [69])
[50]	mPCa	*n* = 27	CellSearch	Binary variable (cutoff: 4)	Yes	CTC enumeration was also predictive of disease progression-free survival
[60]	PCa	*n* = 202	CellSearch	Binary variable (cutoff: 5)	Yes	-
[49]	CRPC	*n* = 55	CellSearch	Binary variable (cutoff: 5)	Yes	Best calculated cutoff to predict overall survival: 3 CTCs/7.5 mL)
				Continuous variable	Yes	
[66]	CRPC	*n* = 57	CellSearch	Binary variable (cutoff: 5)	Yes Independently	-
[61]	CRPC	*n* = 238	CellSearch	Binary variable (cutoff: 5)	Yes Independently	Part of the SWOG SO42 trial
[65]	Previously docetaxel-treated CRPC	*n* = 711	CellSearch	Binary variable (cutoff: 5)	Yes Independently	Part of the COO-AA-301 trial Combination of CTC counts with LDH levels was also a good predictor of overall survival

CRPC: castration-resistant PCa; CTC: circulating tumor cell; FACS: Fluorescence-Activated Cell Sorting; IM: immunomagnetic separation; mPCa: metastatic PCa; PCa: prostate cancer.

### 2.4. Molecular Characterization of Prostate CTCs and Prediction of Treatment Response

Some CTC detection platforms allow partial functional characterization of CTCs, such as the ELISPOT method, which is based on the ability of viable CTCs to secrete particular proteins [41] or the cell-adhesion matrix platform that selects invasive CTCs because of their ability to invade collagenous matrices [13,42]. Overall, recent studies tend to characterize CTCs at the molecular level rather than only enumerate them. Both targeted and genome-wide analyses confirmed clear heterogeneity among prostate CTCs [40] even if comparison with primitive tumor tissues obtained at the time of diagnosis and prior to any cancer therapy disclosed similarities indicative of a clear relationship [77,78]. CTCs can even resemble CRPC tissues taken at autopsy even at the epigenetic level (DNA methylation status at CpG sites) [13]. Such studies allow for identification of putative biomarkers and actors of the metastatic process. As an example, from mRNA-Seq procedures in 67 CTCs from 13 PCa patients, 181 genes overexpressed in CTCs compared to normal prostate tissues were identified that are associated with several biological processes such as metabolic processes, cell cycle or activated AR pathway, providing consistent demonstration of the power of these innovative techniques in the field of the CTCs [79]. Similarly, Smirnov *et al.* [80] could therefore identify a gene set specific for CTCs (whatever the primary cancer tissue), which was evaluated for its ability to discriminate between 74 patients with metastatic disease and 50 healthy donors. Meaningful information may also be provided regarding the metastatic process itself. Schmidt *et al.* [81] evaluated loss of heterozygosity (LOH) patterns in CTCs from 20 patients with multifocal PCas using nine polymorphic microsatellite markers. They also performed the genetic profiling for each individual tumor focus of the prostate. In 17 of the 20 patients, the LOH pattern of the CTCs was identical with only one focus of the primary tumor confirming the well-known molecular heterogeneity of the different foci [82] and the corresponding heterogeneity of the metastatic ability [81]. Of interest, according to the LOH patterns, in six cases, the delivering foci were probably the smaller ones (down to 0.2 mL), providing data to interpret the frequently-observed lack of correlation between the CTC counts and indirect markers of tumor volume such as serum PSA levels [81].

Several studies specifically targeted genes of interest when attempting to characterize CTCs. EMT is considered as a hallmark of migrating and circulating tumor cells (see above). Chen *et al.* [15] obtained 38 CTCs from eight PCa patients they used to determine expression profile of 84 EMT-related and reference genes using a multiplex RT-PCR device. Despite heterogeneous expression patterns, overexpression of genes promoting EMT was commonly observed confirming the loss of epithelial markers and the gain in EMT markers as well as the gain in stemness markers. Some EMT-related genes (PTRRN2, ALDH1, ESR2 and wnt5A) were more expressed in CTCs from CRPC than in CTCs from castration-sensitive cancers [15]. Similar prognostic correlations were identified when studying gene hypermethylation in CTCs from 76 CRPC patients: not only CTC enumeration (binary classification using a cutoff of 5 CTCs/7.5 mL) but also the presence of hypermethylation of five targeted genes were significantly correlated with overall survival [67]. Although correlated to CTC enumeration, the hypermethylation status proved to give significant additional information to predict the patient outcome, underlying the potential clinical benefit of specific molecular phenotyping of CTCs [67].

The fusions that involve the ERG gene (such as the TMPRSS2:ERG fusion) and other members of the ETS transcription factor family have been extensively studied in PCa and proved to play an important role in prostate carcinogenesis [83]. ERG rearrangements have been detected in prostate CTCs [62,84,85,86]. In a study, TMPRSS2:ERG fusion was assessed using RT-PCR in 41 patients with CRPC enrolled in a phase II abiraterone acetate trial [84]. TMPRSS2:ERG fusion was detected in 15 patients and was not predictive neither of post-treatment PSA decline nor of overall survival. When available (*n* = 23 patients), concordance between the RT-PCR assays in CTCs and FISH (fluorescent *in situ* hybridization) assays in primary tumors was only 15/23 patients (65%) [84]. This discrepancy is likely to result from technical pitfalls. When comparisons were available using FISH for all the tissues examined, CTCs, metastases and therapy-naïve primary prostate tumor tissues (cores obtained several years before, prior to any treatment) invariably had the same ERG gene status suggesting that (1) prostate biopsies are able to detect the tumor foci that result in blood-borne metastases, and (2) rearrangement of ERG gene may be an early event in prostate carcinogenesis [62]. By contrast, a significant heterogeneity was observed at the PTEN and AR loci suggesting that these two other key events of the prostate carcinogenesis may occur at different times of the tumor progression or may vary depending on the different treatments administered [62]. Accordingly, Todenhofer *et al.* [24] demonstrated that some CTCs can express markers of EMT or stemness, particularly under docetaxel-based treatment.

Whether CTC molecular phenotyping may serve as a means to predict response to treatment is of great hope for clinicians. While androgen depletion therapy is the first-choice treatment for advanced PCas, the early detection of castration resistance in routine practice is hardly obtained. Whether evaluation of the AR pathway in CTCs could help in predicting response to androgen deprivation is therefore of major importance. In 2012, Myamoto *et al.* [87] classified CTCs obtained from PCa patients as “AR-on” or “AR-off” whether CTCs expressed PSA without PSMA (AR-on) or PSMA without PSA (AR-off). The coexpression of PSA and PSMA corresponded to an “AR-mixed” status. A switch from “AR-off” status to “AR-on” status was frequently observed in metastatic castration-sensitive PCa after one month of androgen deprivation therapy [87]. These results strongly support a role of AR phenotyping in CTCs of PCa patients. Among the various molecular mechanisms that explain castration resistance, mutations in the androgen receptor (AR) have been identified that allow either constitutive (ligand-independent) AR activation or AR activation by extra-gonadal androgens, non-androgenic steroidal ligands or even anti-androgenic drugs. In the CTCs of 35 patients with metastatic CRPCs, Jiang *et al.* [88] searched for AR mutations by RNA amplification followed by complete AR sequence sequencing. Twenty-seven AR mutations were detected in 20 patients, most of these mutations already known to accompany castration resistance [88]. Another study confirmed the possibility to detect AR mutation in prostate CTCs, although with a lesser frequency: two mutations in two out of 37 patients with CRPC [89]. Similarly, AR gene amplification—another mechanism of castration resistance—was also observed in the CTCs of patients with CRPCs [62,78,84,90] Whether AR amplification or mutations could be used in clinical practice as a reflection of castration resistance remains to be determined. Of interest, CTCs have also been evaluated as a marker of resistance to new anti-androgen therapies, such as enzalutamide (inhibition of androgen signaling by competing with and displacing the natural AR ligands) and abiraterone (depletion of adrenal and intratumoral androgens through inhibition of cytochrome P450 17A1). In published clinical trials, 20%–40% of the patients with CRPC have no response to these drugs. It has been hypothesized that such resistances may involve the presence of AR constitutively-active splice variants. Androgen receptor splice variant 7 (AR-v7) is a truncated AR protein that lacks the C-terminal ligand binding domain but retains the transactivating N-terminal domain. It proved to be functional, behaving as a constitutively-active transcription factor in a ligand-independent manner. It can be considered as procarcinogenetic, inducing transcription of a specific transcriptional program, including EMT-related genes [91]. In an elegant, pioneering work, Antonarakis *et al.* [92] evaluated AR-v7 in CTCs from 62 patients receiving enzalutamide or abiraterone. Patients were then classified as AR-v7-positive or AR-v7-negative whether the AR-v7 mRNA was detected or not in the CTCs by RT-PCR experiments. Overall, 39% and 19% of the 31 enzalutamide- and the 31 abiraterone-treated patients had detectable AR-v7 in CTCs, respectively [92]. Similar results were obtained in another study evaluating 47 patients with PSA progression, irrespective of type, line, or sequence of prior therapy (including androgen-deprivation therapies), provided they awaited a therapy switch: 18 of the 37 patients with CTCs had AR-v7 [89]. Of note, AR mutations could simultaneously be detected (using RT-PCR in a distinct fraction of the same CTC samples), demonstrating the ability to determine two key resistance-mediating AR modifications in CTCs. It was demonstrated that AR-v7-positive CTCs were significantly associated with shorter PSA response rate, PSA progression-free survival, clinical progression-free survival and overall survival [92]. AR-v7-positive CTCs were also correlated with the metastatic status [89]. It is worth noting that none of the therapy-naive patients was AR-v7-positive while the presence of AR-v7 correlated with prior treatments [89]. In addition, conversion from AR-v7-negative status to AR-v7-positive status was observed under enzalutamide or abiraterone treatment [92]. Only three out of the seven patients in whom AR-v7 evaluation was available in both CTCs and prostate tissues had simultaneous presence of AR-v7 in CTCs and prostate tissues [92]. Altogether, these results suggest that the presence of AR-v7 is at least revealed or even induced by these treatments [89]. Consistently, it has been demonstrated that treatment of PCa cell lines with either enzalutamide or abiraterone increased the expression of constitutively-active AR-variants, including AR-v7 [93]. Whether the presence of AR-v7 could also predict resistance to taxane therapy remains a matter of debate. Taxanes exert their cytolytic activity by stabilizing microtubules polymers [94,95]. Accordingly, evaluation of microtubule bundling in CTCs of PCa patients has been proposed to estimate response to docetaxel [94]. The microtubule network of prostate cells is also critical for AR translocation from the cytosol to the nucleus and therefore AR transcriptional activity. A correlation has been therefore found between the AR cytoplasmic sequestration in CTCs of patients with CRPC and the clinical response to docetaxel [95]. Mutant deletion studies demonstrated that AR-v7 lacks the microtubule-binding domain of AR [96,97]. Nuclear translocation and transcriptional activity of AR-v7 seems therefore unaffected by taxane treatment [96,97]. Two recent clinical studies however failed to demonstrate an association between the presence of AR-v7 and resistance to taxane therapy [98,99]. Conversion from AR-v7-positive status to AR-v7-negative status was even observed under taxane treatment [100].

### 2.5. Circulating Tumor Emboli (CTC Clusters or Aggregates)

Several studies reported that aggregations of CTCs can also been found in peripheral blood samples of cancer patients. They are considered when at least two or three CTCs are detected together [13,101,102]. Clusters usually contain 4–12 cells (microclusters) [55], but macroclusters composed of up to 100 cells have also been identified [37]. They are named circulating tumor emboli (CTE) or CTC aggregates or clusters and, by comparison with CTCs, have been poorly investigated. Whether circulating clustered cancer cells are technical artifacts has even been questioned [25]. There are, however, several arguments to suggest that CTC clusters are of natural occurrence, such as the lack of observation of cell aggregates in cell line spike-in experiments [55,101], the preserved shape and orientation of tumor cells within the cluster [55], the lack of correlation between the presence of clusters and the absolute number of CTCs in clinical samples [13,55], and the oligoclonal origin of the individual cells within the cluster [103]. It remains nevertheless unknown if they represent real tumor emboli (real detachment of cell aggregates from the primary tumor) or a product of intravascular proliferation [37,104]. For the latter hypothesis, it is also undetermined if cell proliferation occurs during the travel in the bloodstream or when a single cell has attached to the endothelium [104,105].

The currently used technologies for CTC enrichment can detect CTC clusters but they are mostly designed to identify isolate CTCs. The most used technique, *i.e.*, CellSearch system, for example, does not have the ability to isolate CTC clusters. Recent efforts have, however, been made to develop specific microfluidics- or flow cytometry-based methods, the results of which are likely to induce a definitive consensus for a natural occurrence instead of technical artifacts [106,107]. It remains however difficult to assess with precision their true prevalence. Several studies reported CTC clusters in the peripheral blood of PCa patients and the CTC cluster detection rate ranges from 17% to 98% [13,37,55,101,107,108,109,110,111]. These discrepancies are likely to result from technical reasons. Of interest, in an individual study (therefore using a unique method), PCa was found to be more frequently associated with circulating CTC clusters when compared to other cancers, even those known to be frequently metastatic such as the breast and pancreatic ones [101]. Whether PCa exhibits specific characteristics that favor CTC seeding as clusters remains nevertheless to be determined.

Studies for breast, lung and colorectal cancers suggested that the presence of CTC clusters may be indicative of a poor prognosis [102,103,112,113]. To our knowledge, no clear (and CTC-independent) predictive ability has been advocated for CTC clusters in PCa to date but the aggregation of tumor cells in the bloodstream is likely to provide cells with survival advantages. By maintaining cell–cell contacts clustered CTCs resist anoikis. Furthermore, clusters are not always exclusively composed of epithelial tumor cells (homotypic clusters): CTCs can aggregate with other tumor-originating cells such as stromal cells or even cancer-associated fibroblasts (CAF) [114,115] as well as host-derived cells such as hematopoietic and endothelial cells. Experimental data demonstrated that CTC clusters had a higher metastatic potential than single cells [116]. A survival advantage is advocated because of avoidance of anoikis, presence of stromal cells providing a kind of ‘travelling niche’, induction of EMT (by platelets in particular) or avoidance of immune surveillance through incorporation of host cells [2,117]. Circulating tumor microemboli seem therefore to reproduce the functional heterogeneity of the primary tumor [118].

## 3. Other Circulating Tumor Cellular Materials

CTCs are not the only circulating tumor materials. Many other materials can be found that result either from (1) processes that destruct (circulating or not circulating) tumor cells or (2) processes used by viable tumor cells to deliver messages to other cells. In the first case, as much as half of the circulating cells are thought to perish within 2.4 h following their introduction into blood circulation [5]. The various destructive processes (apoptosis, necrosis, cytolysis, *etc*.) that cause CTC death result in leakage of intracellular components (such as nucleic acids) through perforations of the CTC membranes and circulation of cellular debris or damaged or fragmented cells. In the second case, there is growing evidence that (normal and cancer) cells produce diverse extra-cellular vesicles, which are informative because of their membranous structure (composed of various lipids) and their own contents. Such extra-cellular vesicles now clearly appear as messengers used for cell-to-cell communication.

When considering all these destructive or productive mechanisms, it becomes evident that the strict criteria used for defining CTCs by the CellSearch system obligatory result in neglecting other EpCAM-positive objects. When applying the strict definition of CTCs, 23% of the cancer samples were considered as negative for CTCs, although several of them contained in fact other EpCAM-positive objects [76]. This could explain why some CTC-negative patients may eventually have a bad outcome. Consistently, not only the CTC count as defined by CellSearch criteria but also all classes of EpCAM-positive objects were found to be predictor for overall survival, suggesting that (1) other tumor materials, parts of tumor cells, may circulate in a manner similar to true CTCs, and (2) further studies are needed to examine which class (or which ratio to one another) is the most suitable to evaluate the prognosis [76].

### 3.1. Circulating Vesicles

Extracellular vesicles (EVs) are heterogeneous populations of vesicles delimitated by a cell membrane and released by cells into their microenvironment and blood circulation [119]. Recent studies provided valuable data about the role of EVs in many physiological, pathological, diagnostic and therapeutic aspects such as intrinsic normal cell biology, pathogenesis, drug, vaccine and gene-vector delivery, and as possible reservoirs of biomarkers. Overall, they are thought to mediate the exchange of intercellular messages (their “cargo”) comprised of various assembled bioactive molecules including classical factors, structural proteins, nucleic acids and lipids: intercellular “signalosomes” [120]. They can *in vivo* travel in all body fluids to distant sites, behaving therefore like endocrine factors when circulating in the bloodstream [119]. A specificity of EV production by cancer cells is indeed the intercellular trafficking of bioactive molecules containing oncogenic mutations, such as activated oncoproteins, their transcripts, oncogenic DNA sequences as well as regulatory micro RNA (in this context, EVs are therefore called oncosomes) [121]. It has been clearly demonstrated that the uptake of this transforming cargo by recipient cells cause changes in their phenotype and biological behavior [122]. By a mirror effect, host cells activated because of the cancer presence (macrophages, leucocytes, platelets, endothelial cells, bone marrow progenitors, *etc*.) also produce specific EVs [123]. Besides the considerable hope raised from the numerous studies published about EVs and their roles in physiology and pathology, it should always be kept in mind that there is no real, widely-accepted nomenclature of EVs [124] even if an expert panel recently proposed to distinguish apoptotic bodies, microparticles (or microvesicles) and exosomes, as the three main classes, depending on their size and their biogenesis (Table 5) [125]. Another great difficulty is the limitation in selectively differentiating one EV population from another because current methods of purification often result in mixtures of particles [119].

**Table 5:** The three main classes of extracellular vesicles, according to [125].

Extracellular Vesicles	Size Range	Production	Cell of Origin	Markers
Apoptotic bodies	0.5–5 mm	During the late stage of apoptosis	All cell types	Expression of phosphatidylserine on the membrane surface
Microvesicles (or microparticles or ectosomes)	0.2–1 mm	Outward protrusion/budding from the plasma membrane	Tumor cells Polynuclear leukocytes Aging erythrocytes	Expression of phosphatidylserine on the membrane surface
Exosomes	40–100 nm	Endosome-derived Liberation by fusion with the plasma membrane	Probably all cell types	Alix TSG101 Tetraspanins Heat shock proteins

Prostate exosomes are by far the most frequently studied EVs. They are known for a long time and were essentially studies in the semen as specific organelles expressed in prostatic secretions [126]. They can be called “prostasomes” but this term is classically reserved for seminal (or urinary) prostate exosomes detected or analyzed in the prostate secretions. The recent enthusiasm for cancer exosomes also affected PCa with several works dealing with proteomics, genomics or lipidomics of PCa cell line-derived exosomes in an attempt to identify, through high throughput methods, potential biomarkers (review in [127,128]). Bijnsdorp *et al.* [129] identified exosomal ITGB1 and ITGA3 by proteomics analysis of exosomes derived from PC3 and LNCaP cells. They found that inhibition of exosomal ITGA3 reversed the effect of PC3- and LNCaP-exosomes on migration and invasivity of PrEC. Moreover, ITGB1 and ITGA3 were more abundant in urines of patients with metastatic PCa than in patients with BPH, confirming the ability of exosome studies to identify potential actors and markers of prostate carcinogenesis [129]. For prostate exosome-based clinical studies, urine is the most used body fluid and several data are available since the first one, which demonstrated both feasibility and sensitivity of exosomal detection by amplifying exosomal RNAs of two known PCa markers: PCA3 and TMPRSS2:ERG fusion [130]. Detection of prostate-derived exosomes circulating in the bloodstream also proved to be feasible [131]. Levels of circulating exosomes were indeed measured in plasma derived from 16 healthy controls, 20 patients with prostate benign hypertrophy (PBH), and 47 PCa patients (including eight taxotere-resistant patients) [132]. Exosomes were purified by differential centrifugation and exosomal survivin was then measured by Western blot. Overall, exosome levels and exosomal survivin were found to be higher in PCa patients than in healthy controls and PBH patients [132]. No difference were observed depending on the pre-treatment Gleason score (10 patients with Gleason 6 *vs.* 10 patients with Gleason 9). By contrast, patients with taxotere-resistant PCa had higher exosome levels and exosomal survivin than other PCa patients [132]. Similarly, plasma exosomes were isolated in plasma of five PCa patients using anti-PSMA magnetic beads and quantified using the exosomal marker CD9. CD9-expressing exosomes were more numerous in the three patients with metastatic or chemoresistant PCa than in the two patients with non-metastatic PCa [133]. Higher exosome levels in PCa patients than in healthy controls were also observed in another study, with a positive correlation with the Gleason score [134]. Further studies, including larger cohorts, are warranted to explore these issues for routine applications.

Studies about EVs in PCa essentially focused on exosomes but other EVs have been reported. In cancer, ectosome-like structures may originate from membrane blebs that are associated with the ameboid motility of certain types of tumor cells (motility different from the mesenchymal fibroblast-like EMT-related mode, rather resembling that of amoebae with poor adherent properties and extensive membrane deformation). These abnormal EVs are large enough to be observed by light microscopy and are referred to as large oncosomes [135]. In a murine model of metastatic PCa, they proved to be quantified in tissues but also in plasma where they have been associated with PCa aggressiveness [135]. A recent proteomics study found that proteins are differently represented in large *vs.* nanosized EVs from PCa cells [136]. Large oncosomes are particularly enriched in enzymes involved in glucose, glutamine and amino acid metabolism, metabolic processes able to induce alterations of the glutamine metabolism of cancer cells [136]. Further studies are required to determine the extent of the overlap between large and small vesicles in terms of molecular cargo and function [119]. In clinical practice, large oncosomes can be detected in the plasma and the presence of caveolin-1 in them has been found to correlate with metastatic disease [137]. Like other EVs, large oncosomes also proved to mediate intercellular transfer of functional microRNAs [137], which, by themselves, can be detected as circulating tumor materials.

### 3.2. Circulating Nucleic Acids

Several nucleic acids can be found as circulating materials in the peripheral blood. They include DNAs, mRNAs, microRNAs and long non coding RNAs. Whether the nucleic acids identified in the serum or the plasma originate from a tumor can indeed only be ascertained when they contain tumor-specific somatic alterations. The corresponding advantage is that these alterations constitute extremely specific biomarkers for cancer that can be easily detected with the appropriate technique and tracked over time. It is, however, very challenging to detect tumor-originating circulating acids since they are surrounded by multiples copies of normal nucleic acids. Mostly, these nucleic acids can circulate under various conditions. Circulating DNA is mostly found free of any cell or any cell fragment (cell-free DNA: cfDNA), while RNAs are essentially considered as part of the exosomal content.

#### 3.2.1. Circulating DNA

Circulating DNA is usually released as small fragments (150–200 bp in length [138]) from normal or tumor cells by apoptosis or necrosis [139]. The balance between a necrotic and an apoptotic pattern seems to be different among the various cancers: by contrast to colon or breast cancer (where larger fragments suggest a predominant necrotic breakdown), short DNA fragments are detected in PCa and suggest a predominant apoptotic release [140,141]. Circulating tumor DNA comprises between 0.01% and 90% of cfDNA [142].

It was found as soon as 1977 that serum circulating cfDNA levels were higher in cancer patients than in non-cancer patients [143]. Since circulating tumor DNA fragments are theoretically released from all tumor sites (primary tumors, lymph node metastases, distant metastases) and from all parts of each tumor site, it can be considered as a reliable witness of the whole tumor burden and can therefore be used as a means to monitor it under treatment. As such, it constitutes a kind of repeatable and easily available liquid biopsy. The correlations found between the presence of CTCs and the detection of circulating tumor DNA [144] and the possible higher sensitivity of circulating DNA genotyping over the direct detection of CTCs [145,146,147] reinforce its potential interest as the ideal biomarker. Of note, discordances between the genetic aberrations of the primary tumor and the alterations of the circulating DNA in blood have been described for several cancers and suggest that (1) cfDNA can be a biomarker of tumor cell dissemination and that (2) the “parallel development” hypothesis (primary tumor and secondary sites develop similar genetic alterations in a parallel way) remains to be demonstrated [148].

Cell-free DNA can be extracted from serum or plasma using commercial kits. Of note, for yet unknown reasons, DNA levels are largely higher (about six-folds) in serum than plasma, even if the lack of a standardized processing procedure make comparisons difficult [140]. PCR-based methods are the most currently used but usually require the choice of the genetic alterations to be detected. Other methods, notably high throughput ones, are under development that allow direct massive parallel sequencing of the whole circulating DNA populations [149]. There are in fact variations concerning what is really evaluated: whole cfDNA concentration, targeted genetic aberrations, whole DNA genetic aberrations, cfDNA integrity of epigenetic events such as hypermethylation.

Table 6 provides summaries of the published studies that assessed serum or plasma cfDNA levels and compared them according to the presence or the absence of PCa.

Overall, conclusions can hardly be drawn because of the extreme variation in the designs of the 19 available studies. The control groups, for example, consisted of either healthy controls, patients with negative biopsies, BPH patients, prostatitis patients or a mix of these conditions. PCa patients were also highly heterogeneous including various stages and various combinations of stages. This is likely to be of importance since one study reported higher cfDNA levels in metastatic patients when compared to healthy controls and no difference between patients with localized PCa and healthy controls [144]. This probably explains the contradictory results with several studies being unable to demonstrate any difference between PCa patients and controls [144,150,151,152,153] while others did [154,155,156,157,158,159,160,161,162,163]. Whether cfDNA levels could be used for prognostic purpose also remains to be determined (Table 6). Only two studies disclosed correlation with Gleason score [156,160] while six others did not [141,152,153,155,161,163]. Similarly, a correlation with pT stage was reported [155,156] or not [150,164]. While failing to identify correlations with Gleason score or pT stage, Jung *et al.* [152] observed a correlation between cfDNA levels and overall survival. cfDNA levels were found to predict biochemical recurrence in localized PCa after radical prostatectomy [156]. Whether cfDNA could be used as a predictor or a marker of evolution under treatment is also suggested by a recent study that included eight CRPC patients submitted to docetaxel [164]. In this study, tumor activity on PET/CT correlated with cfDNA levels at baseline and patients with criteria for PET tumor response under treatment had significantly lower pretreatment cfDNA levels than those who did not. Of interest, an increase in cfDNA levels was observed after docetaxel treatment, along with the appearance of large fragments suggestive of necrosis [164], suggesting the ability of cfDNA levels to monitor treatment action. A similar increase in cfDNA levels has also been reported after surgery or androgen deprivation [141,154].

Measuring circulating levels is not the only way to evaluate cfDNA in blood. Whether circulating DNAs are intact or not seems also to be informative [150,165,166]. Several studies also demonstrated the presence of genetic instability (microsatellite instability), specific genetic alterations (mutations) and epigenetic alterations (promoter hypermethylations) [147]. The analysis of allelic imbalance is feasible in circulating DNA and the presence of microsatellite instability appeared highly specific (specificity: 70%–100%) of the presence of PCa in all six studies that evaluated it [144,167,168,169,170,171]. Direct identification of specific genetic alterations seems also promising, as suggested by two recent studies. Azad *et al.* [172] evaluated discovered several AR gene aberrations in circulating cfDNA of 62 patients with progressive metastatic CRPC after various treatments including abiraterone and enzalutamide. These AR gene alterations (mutations, variation in copy number, *etc*.) could be therefore tested as biomarkers of treatment resistance in CRPC. Similarly, copy number variations in circulating AR and CYP17A1 DNAs proved to correlate with progression-free survival (as assessed by PSA dynamics) and overall survival [173]. Epigenetic alterations in circulating cfDNA have been more intensively explored in PCa patients (Table 7). Eighteen studies are available, including 11 that dealt with hypermethylation of the GSTP1 promoter. This epigenetic alteration is indeed frequently observed in prostate tumors and can also be detected in tissue and urine samples. Its detection in circulating DNA has been reported as soon as 2000 [174], with a consistent high specificity [153,170,174,175,176,177,178,179]. Other promoters have been hypermethylated in circulating DNA, including CD44 [180], AR [178], MDR1 [181], RARβ [170,179,181], TIG [177], RASSF1 [170,179], APC [179], Gal3 [182], histone H3 [183], CDH13 [184] and Gadd45a [185]. A global profiling using microarrays allowed identification of the gene RNF2019 as a target for hypermethylation in PCa when compared to healthy controls [186]. Whether hypermethylated promoters found into the circulating DNA could be used as prognostic markers remains to be determined since contradictory results have been found in relation to either Gleason score or pT stage [146,170,177,178,180]. Promising correlations with disease progression [179], biochemical recurrence [175] or even overall survival [184] have been reported but need confirmation by further studies.

**Table 6:** Diagnostic and prognostic information of serum and plasma cell-free DNA levels in prostate cancer.

Reference	Number of PCa	Number of Controls	Fluid	Method	Results for Circulating DNA Levels
[152]	91	34 BPH 59 healthy controls	P	FA	No difference between lPCas and controls N1M1 PCa and BPH Patients with mPCa under ADT or not No correlation between DNA levels and PSA, pT, Gleason in pN0M0 PCa Correlation between DNA levels and PSA in M1 PCas Correlation between DNA levels and overall survival
[187]	12	13	P	RT-PCR	Se = 58%; Spe = 92%; AUC = 0.708
[154]	15	10 BPH 12 HGPIN	P	RT-PCR	Increase in DNA levels after prostate biopsies No difference between PCa and HGPIN When comparing PCa + HGPIN *vs.* BPH: Se = 85% ; Spe = 73%
[157]	78	15 patients with low PCa risk ^a^ 74 patients with negative biopsies 10 healthy controls	P	RT-PCR	Increase in DNA levels in PCa *vs.* the 15 patients with negative biopsies and the 10 healthy controls Increase in DNA levels in the 74 with negative biopsies *vs.* the 78 PCa patients
[153]	12 newly diagnosed PCa 15 PCa subjected to treatment	13 healthy controls	P	RT-PCR	Increase in DNA levels in newly diagnosed PCa *vs.* the healthy controls No difference in PCa patients subjected to treatment and healthy controls No correlation between DNA levels and Gleason score
[150]	61	62	P	RT-PCR	No difference between the two groups
[159]	142 lPCa	19 BPH	P	SA	Increase in DNA levels in PCa patients Increase in predictive accuracy when DNA levels are added to a base model
[156]	192 lPCa 18 mPCa	35 patients with negative biopsies	S	RT-PCR	Increase in DNA levels in: mPCa patients *vs.* the lPCa patients lPCa patients with PSA recurrence *vs.* lPCA without PSA recurrence In lPCa patients, correlation between DNA levels and Gleason at biopsy, Gleason at prostatectomy, positive surgical margins and pT
[140,177]	168 lPCa 5 incidental PCa ^b^	42 BPH 11 healthy controls	S	RT-PCR	When comparing the 168 lPCa to the 42 BPH : Se = 88%, Spe = 64% and AUC = 0.824
[155]	64	45 healthy controls	P	RT-PCR	Increase in DNA levels in PCa patients Se = 80%, Spe = 82% and AUC = 0.881 Correlation between DNA levels and pT No correlation between DNA levels and Gleason or PSA
[151]	5	22 BPH 30 healthy controls	P	FA	No difference between PCa and BPH
[144]	69 lPCa 12 mPCa	10 healthy controls	P	SA	Increase in DNA levels: in mPCa patients *vs.* lPCa patients in mPCa patients *vs.* healthy controls No difference between lPCa patients and healthy controls
[161]	89	104 BPH 59 prostatitis	S	RT-PCR	Increase in DNA levels: in PCa patients *vs.* BPH patients in PCa patients *vs.* BPH and prostatitis patients No difference between BPH patients and prostatitis patients No correlation between DNA levels and Gleason score Increase in predictive accuracy when DNA levels are added to a base model
[164]	8 CRPC	-	P	RT-PCR	Increase in DNA levels after docetaxel therapy No correlation between DNA levels and PSA Correlation between DNA levels and tumor activity at PET/CT imaging
[141]	19	20 healthy controls	P	RT-PCR	Increase in DNA levels after 3 month ADT or 3 months after surgery No correlation between DNA levels and Gleason, PSA doubling time or PSA recurrence
[160]	96	112 BPH	P	RT-PCR	Increase in DNA levels in PCa patients Correlation between DNA levels and PSA or Gleason
[163]	133	33 patients with negative biopsies	P	SA	Increase in DNA levels in PCa patients Se = 66%, Spe = 88% No correlation between DNA levels and PSA, Gleason, pT, or BRFS Correlation between an increase in DNA levels during the follow up (sampling every 3 months during 2 years) and BRFS
[162] ^c^	85	101 BPH 55 prostatitis	S	FA	Increase in DNA levels in PCa patients Increase in predictive accuracy when DNA levels are added to a base model
[158]	16	25 BPH 40 healthy controls	P	FA	No difference between PCa and BPH Increase in the ratio cell-free/total circulating DNA in PCa and BPH patients *vs.* healthy controls

^a^ negative prostate biopsies two years ago and normal PSA velocity within the last 2 years; ^b^ PCa discovered in TURP specimen; ^c^ patients extracted from [161]; ADT: androgen-deprivation therapy; AUC: area under ROC curve; BPH: benign prostate hyperplasia; BRFS: biochemical recurrence-free survival; CRPC: castration-resistant prostate cancer; FA: fluorometric assay; HGPIN: high grade prostate intraepithelial neoplasia; lPCa: localized PCa, mPCa: metastatic PCa; P: plasma; PCa: prostate cancer; PSA: prostate-specific antigen; RT-PCR: reverse transcriptase-polymerase chain reaction; S: serum; SA: spectrophotometric assay; Se: sensitivity; Spe: specificity; TURP: trans-uretral resection of the prostate.

**Table 7:** Diagnostic and prognostic information of methylation status of cell-free DNAs in prostate cancer.

Reference	Number of PCa	Number of Controls	Fluid	Method	Studied Gene(s)	Results			
[174]	33	26 BPH	S & P	MSP	GSTP1	Se = 72%; Spe = 100%			
[180]	7	-	S	MSP	CD44	Se = 100% No correlation with the pM status Physiologic hypermethylation in several normal epithelia			
[175]	85 lPCa 18 CRPC	35 patients with negative biopsies	S	qMSP	GSTP1	Spe = 100% Se = 12% in lPCa patients and 28% in CRPC (*p* = 0.003) Correlation with biochemical recurrence and BRFS			
	110 patients with RP 55 with recurrence 55 without recurrence	-	S	qMSP	GSTP1	GSTP1 methylation in 8 patients with recurrence and none of the patients without recurrence			
[153]	31	9 healthy controls	P	MSP	GSTP1	Se = 52%; Spe = 100%			
[178]	14 lPCa 62 CRPC	49 healthy controls	S	MSP	- GSTP1 AR 14-3-3β	Healthy 0 27% 55%	lPCa 21% 36% 86%	CRPC 32% 40% 87%	Significant increase for GSTP1 Correlation between GSTP1 methylation and Gleason, pM and pN status No correlation between GSTP1 and PSA, overall survival, response to treatment
[176]	36	27 BPH	P	MSP	GSTP1	Se = 31% ; Spe = 93%			
[181]	192 lPCa 18 CRPC	35 patients with negative biopsies	S	qMSP	Several genes including MDR1	MDR1 was the only hypermethylated promoter in lPCa: 16% of the patients without biochemical recurrence 38% of the patients with biochemical recurrence IN CRPC patients, hypermethylation in MDR1 (89%), EDNRB (50%), RARβ (39%)			
[188]	5	5 BPH 5 healthy controls	P	MSP and sequencing	GSTP1	Sequencing provided different methylation patterns according to pathological diagnosis.			
[177]	168 PCa 5 incidental PCa	42 BPH 11 healthy controls	S	MSP	- GSTP1 TIG1 PTGS2 Reprimo	BPH 8% 0 0 0	PCa 42% 10% 2% 1%	GSTP1 methylation in 4 of the 5 incidental PCas Significant difference between BPH and PCa for GSTP1 and TIG1 No correlation between methylation and pT, Gleason or biochemical recurrence	
[179]	20 PCa with disease progression 22 PCa without disease progression	22 BPH	Whole blood	qMSP	Several including GSTP1 RASSF1a APC RARβ	BPH 9% 23% 9% 9%	Not recurring PCa 91% 95% 91% 68%	Recurring PCa 100% 100% 95% 90%	Significant increase with PCa and disease progression.
[182]	2 PCa stage II 1 PCa stage III 1 PCa stage IV	1 BPH	S	MSP	Gal3	No Gal3 hypermethylation on the BPH patient and in the stage III and IV patients The 2 patients with stage II PCa exhibited Gal3 hypermethylation			
[170]	83	40 healthy	S	MSP	GSTP1 RASSF1 RARβ2	12% 24% 13%	None of the healthy controls exhibited hypermethylation At least one hypermethylation in 28% of the PCa patients Correlation between the presence of at least one hypermethylation and PSA, Gleason score and stage		
[183]	22 lPCa 11 locally advanced PCa 28 mPCa	-	P	ELISA	H3K27me3 (trimethylated histone H3 lysine 27)	The median plasma level of H3K27me3 was significantly lower in mPCa than in lPCa and locally advanced PCa			
[186]	19 PCa	20 BPH 20 healthy controls	P	Micro-array	Global profiling	In this exploratory set, no difference in the methylation patterns between PCa and BPH 39 PCa-associated changes when compared to healthy controls; 7 out of them were confirmed by sequencing, including RNF219 Diagnostic performances of RNF219: Se = 89%, Spe = 71%, AUC = 0.79			
	20 Pca	18 BPH	P	PS	RNF219	In this validation set, the diagnostic performances of RNF219: Se = 61%, Spe = 71%, AUC = 0.56			
[146]	75 CRPC before chemotherapy	-	P	qMSP	GSTP1	No correlation with Gleason score, bone metastasis status or PSA response to treatment GSTP1 hypermethylation was an independent predictor of overall survival Correlation between methylated GSTP1 levels after the first chemotherapy cycle and PSA progression			
[184]	98	27 BPH 9 healthy controls 11 bladder stone	S	MSP	CDH13	Se = 45%; Spe = 100% Correlation with Gleason score, pT, and PSA CDH13 methylation status was an independent predictor of overall survival			
[189]	694	703	P	PS	Line1 Alu	Iterative samples as part of the Prostate, Lung, Colorectal and Ovarian cancer screening trial No correlation with PCa or PCa aggressiveness Variations were observed for Alu methylation status depending on the time between blood sampling and PCa diagnosis			
[185]	34	48	S	PS	GADD45a	Higher levels in PCa patients No correlation with Gleason score			

AUC: area under ROC curve; BPH: benign prostate hyperplasia; BRFS: biochemical recurrence-free survival; CRPC: castration-resistant prostate cancer; lPCa: localized PCa; mPCa: metastatic PCa; MSP: methylation-specific polymerase chain reaction; P: plasma; PCa: prostate cancer; PS: pyrosequencing; qMSP: quantitative MSP; RP: radical prostatectomy; S: serum; Se: sensitivity; Spe: specificity.

#### 3.2.2. Circulating MicroRNAs

MicroRNAs are small non-coding RNAs. They have been shown to be involved in a range of important regulatory cellular functions. Ubiquitous in all mammalian cells, microRNAs are produced through a complex processing pathway including the action of the RNase III enzyme Dicer and the assembling of the mature microRNA strand into an RNA-induced silencing complex (RISC). This complex permits binding to specific target mRNAs to regulate post-transcriptional gene expression through translational repression and mRNA degradation. MicroRNA binding is dependent on the recognition of two to eight nucleotides (the ‘seed sequence’) at the end of its complementary mRNA target. Perfect base-pair complementary between the microRNA and its target results in cleavage of the target by the argonaute enzyme present in the RISC, while imperfect complementary results in translational repression and degradation of the target. It is worthy to note that there is growing evidence that the microRNA maturation process is linked to the formation and maturation of EVs and particularly to microvesicles and exosomes [190]. It has indeed been demonstrated that pre-miRNAs loaded into the RISC complex may be sorted into late endosomes (precursors of exosomes). It remains to be determined whether sorting of pre-miRNAs into exosomes could also be occurring in a sequence-dependent manner [190]. It is also still of debate whether circulating microRNAs exist as isolated cell-free molecules [191]. In addition to being packed into exosomes or microvesicles, extracellular microRNAs can circulate within high-density lipoprotein (HDL) [192,193] or bound by AGO2 protein outside of vesicles [194].

Aberrant expression of microRNAs (up- or down-regulation) has been observed in a diverse range of pathological conditions (including PCa: [195]) because of chromosomal rearrangements (mutations, deletions, amplifications), promoter methylation, and regulation of expression. A number of techniques for microRNA profiling has been progressively developed that allow precise quantification and identification in both solid tissues and body fluids, with techniques specifically designed towards a microRNA of interest or towards high throughput identification of the whole microRNA repertoire in a clinical condition. PCa also proved to aberrantly express microRNAs (review in [195]) and there is now strong evidence suggesting that deregulation of microRNA expression is involved in both PCa pathogenesis and treatment resistance. Numerous microRNAs have indeed been shown to influence key cellular processes involved in prostate carcinogenesis such as apoptosis-escape, cell proliferation, cell invasion, cell migration, androgen receptor signaling, EMT, immune escape, *etc.* (review in [196] and [197]). Of note, several of the signaling pathways known to be deregulated during prostate carcinogenesis (AR signaling, PTEN/Akt, TMPRSS2:ERG fusions, *etc*.) exert their oncogenic properties at least partly through deregulation of biologically relevant microRNAs [196].

Since the first descriptions of the fact that cell-free microRNAs have potential as noninvasive diagnostic markers in body fluids [198], several studies proved that they are indeed stable (resistance to RNase degradation because of their short sequence length) and reproducibly measurable in plasma and serum [199]. In PCa, Mitchell *et al.* [200] reported in 2008 that several circulating microRNAS, and specially miR-141, were significantly elevated in the sera of PCa patients when compared to healthy controls. This pioneer work was confirmed by others correlating various miRNAs with the presence of PCa or with PCa risk, clinicopathological parameters, PCa aggressiveness, staging, and disease outcome (Table 8). More recently, microRNAs have also been suggested as putative mediators of treatment response [201].

**Table 8:** Diagnostic and prognostic information of circulating microRNAs associated with prostate cancer.

Reference	Number of PCa Patients	Number of Controls	microRNAs Found to be Deregulated in Peripheral Blood	Remarks
[200]	25 patients with mPCa	25 healthy controls	miR-100, -125b, -141, -143, and -296	miR-141 was the most significantly increased
[202]	5 patients with PCa	8 healthy controls	miR-16, -92a, -103, -107, -197, -34b, -328, -485-3p, -486-5p, -92b, -574-3p, -636, -640, -766, ans -885-5p	Several patients were pre-treated with chemotherapy
[203]	36 patients with PCa	12 healthy controls	miR-223, -26b, -30c, -24, -874, -1247a, -1207-5p, -93, and -106a	miR-24 and miR-106a decreased and increased with PCa aggressiveness, respectively
[204]	51 patients with PCa	20 healthy controls	miR-21 and -221	miR-141 was also elevated when considering only mPCa
[205]	21 patients with mPCa	-	miR-141	Correlation with clinical progression and PSA
[206]	50 patients with PCa	6 patients with BPH	miR-21	Elevation only on patients with CRPC and patients with hormone-sensitive mPCa Higher levels in patients with resistance to docetaxel
[207]	21 patients with PCa	-	miR-375, -9*, -141, -200b, and -516a-3p	-
	116 patients with PCa	-	miR-375 and -141	Higher levels in high-risk patients (Gleason score ≥ 8 or metastases) Higher levels of both miR in patients with positive lymph nodes.
[208]	25 Patients with CRPC	25 healthy controls	miR-141, -298, -246, and -375	-
[209]	70 Patients after surgery	-	miR-141, -146b-3p, and -194	Prediction of biochemical resistance following radical prostatectomy
[210]	78 patients with PCa	28 healthy controls	miR-107, -130b, -141, -2110, -301a, -326, -331-3p, -432, -484, -574-3p, -181a-2, and -625	miR were evaluated within circulating exosomes and larger microvesicles Higher levels of miR-221, - 375, and -141 in patients with mPCa as compared to non-metastatic patients
[211]	23 Patients with CRPC	-	miR-375 and -1290	miR were evaluated within circulating exosomes
[212]	84 patients with PCa	-	miR-375, -378, 409-3p, and -141	Higher levels in CRPC patients than in patients with lPCa
[213]	25 patients with PCa	17 patients with BPH	miR-let-7e, -let-7c, -30c, -622, and -1285	-
[214]	82 patients with PCa	-	miR-20a, -21, -145, and -221	Smaller levels in patients with lPCa
[215]	45 patients with PCa	18 patients with BPH and 20 healthy controls	miR-26a, -195, and let-7i	-
[216]	54 patients with positive prostate biopsies	79 patients with negative prostate biopsies	miR-26a-1 and -141	Diagnostic cohort of 133 patients undergoing prostate biopsies No difference in miR levels in the 2 groups Increased levels of miR-141 with increasing Gleason score in patients with positive biopsies
[217]	75 patients with positive prostate biopsies	27 patients with negative prostate biopsies	miR-let7a, -141, -145, and -155	Higher miR-141 levels with d’Amico’s classification
[218]	150 patients with PCa prior to surgery	50 patients with BPH	Combination of expression levels of 14 miRNAs into a “miR Score”	Lower levels in high-risk cancer
[219]	97 patients with CRPC	-	miR-200b and -20a	Correlation with overall survival
[220]	59 patients with PCa	16 patients with BPH and 11 healthy controls	miR-375 and –let-7c	Higher diagnostic performances when the two miR were combined.
[221]	31 patients with PCa	13 patients with BPH	miR-375 and -141	Higher diagnostic performances when the two miR were combined.

BPH: benign prostate hyperplasia; CRPC: castration-resistant prostate cancer; lPCa: localized PCa; mPCa: metastatic PCa; miR: microRNA; PCa: prostate cancer, PSA: prostate-specific antigen.

## 4. Conclusion

The management of PCa patients is currently moving towards personalized medicine, which is the adaptation of treatment (first line or other lines) to the intrinsic molecular biology of the tumors. While new targeted therapies are currently under development, there is a crucial need for reliable tools able to identify and follow, over time, molecular alterations and signaling pathway activations. As a non-invasive and reproducible method, peripheral blood is a near-ideal sampling site. Whether other witnesses of tumor burden than PSA can be drawn from serum and/or plasma is therefore of major importance. In this setting, several circulating tumor materials can be found and have been the targets of numerous experimental and clinical studies. We needed further studies to provide methods standardization, power of large cohorts, and eventually translation towards clinical practice. The recent report of long-term, patient-derived prostate cancer lines obtained from CTCs using a 3D organoid system [86] would provide clinicians with valuable tools for personalized genetic and pharmacological studies.
